# The Performance of Commercial pH‐Sensitive Ion‐Selective Field Effect Transistors

**DOI:** 10.1002/open.202500361

**Published:** 2025-09-24

**Authors:** Nandor Ziebart, Alexander Gießel, Thomas Walther

**Affiliations:** ^1^ Chair of Bioprocess Engineering Technical University of Dresden ZINT Campus 01069 Dresden Germany

**Keywords:** autosamplers, electrochemistry, ion‐selective field effect transistor sensors, pH sensors, sensor evaluation, sensors

## Abstract

pH‐sensitive ion‐selective field effect transistors (ISFETs) are commercially available nowadays, offering high robustness, resolution, accuracy, and durability. A crucial drawback is their high price and permanent power demand. Incorporating commercially available small‐sized ISFET sensors into battery‐powered devices requires a thorough evaluation and the development of strategies to reduce power consumption. In this work, a direct comparison of three different commercial ISFETs is presented using a newly developed evaluation process that aims to investigate long‐term performance. The different behaviors in conditioning, linearity, accuracy, response times, and long‐term stability in detail are discussed. Furthermore, strategies to decrease power consumption are presented by adjusting the operating conditions and introducing an OnOff‐protocol. It is found that all ISFETs have limitations in their overall performance with the best performers being 1) Winsense, with the highest slope of 59.7 mV pH^−1^; 2) Microsens, with the highest potential output precision under the recommended working point conditions (±0.01−0.03 pH) with SD and reduced working conditions (± 0.01 pH) with WD; and 3) Sentron, with the highest stability under both power‐reducing strategies. With the combination of both power‐reducing strategies, the ISFET's power demand is reduced by 98.8%.

## Graphical abstract

Herein, research into the performance of commercially available ion‐selective field effect transistor (ISFET) sensors for long‐term biotechnological applications is presented. An evaluation method, an ISFET OnOff working protocol, and a DIY autosampler are developed and described in detail. ISFET sensors are used at different working point settings, achieving an overall reduction in power demand of 98.8%.

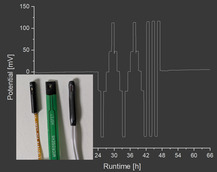

## 1 Introduction

In addition to dissolved oxygen and temperature, the activity of hydrogen ions is one of the parameters of interest in monitoring and control of biotechnological processes. Metal‐oxide layers are well known for their reproducible electrochemical response to analyte concentration. Besides potentiometric applications in ion‐sensitive electrodes (ISEs), a key milestone in the development of selective pH‐sensing materials was the invention of ion‐selective field effect transistor (ISFET) for Na^+^‐ions, which uses an analyte‐sensitive metal–oxide layer. This was first described in 1970 by Bergveld.^[^ ^1^ ^]^

Starting from a new sensor type with many drawbacks, ISFETs have made significant improvements in terms of accuracy, drift reduction, light sensitivity, temperature dependency, and cross‐sensitivity over the decades. The inventor of the ISFETs himself, Piet Bergveld, provided a comprehensive overview of the history of ISFET development, their sensor instrumentation, and ISFET theory in 2003.^[^ ^2^ ^]^

Following initial confusion within the scientific community regarding the sub‐Nernstian potential response behavior of various oxides, a new model was developed for making precise predictions of the behavior of different materials in ISFET applications. The molecular mechanism of the interaction between the hydroxyl groups of the metal oxide and the solution can be described by the site binding theory. First presented in 1973, the model describes the formation of the electrochemical double layer at the interface between the electrolyte and the metal oxide.^[^ ^3^ ^]^ The introduction of the material‐property‐related variable *β*, which describes the oxide layer's buffer capacity, made theoretical calculations of the ISFET potential outcome possible. For a thorough theoretical background with conclusive figures, the 2021 review by Sinha and Pal is highly recommended.^[^ ^4^ ^]^

The sensing technology for gate‐open field‐effect transistors can be characterized by three keyelements: 1) the threshold voltage (*V*_th_ or *V*_ds_) between drain and source, which allows a current to flow through the transistor in the measurement mode; 2) the drain‐source current (*I*_ds_) resulting from the applied *V*_ds_ must be kept within the nonsaturated linear conducting region of the transistor; and 3) the gate‐source threshold voltage (*V*_gs_), which rises with the analyte concentration‐dependent polarization of the gate layer.

A broad explanation of the working principle and a comparison with MOSFET‐technology (metal–oxide‐semiconductor field effect transistor) can be found in the 2023 review by Hassan et al.^[^ ^5^ ^]^ All of the commercially obtained ISFETs used in this article employ potentiometric technology, which involves keeping *I*_ds_ and *V*_ds_ constant (also known as constant voltage–constant current (CVCC) mode), and measuring the proton activity that alters the gate–source voltage *V*_gs_.

ISFETs have several advantages for parallelized biotechnology applications, such as being manufactured with standard metal–oxide semiconductor processes, which makes them mass produceable. Furthermore, they are suitable for continuous monitoring, and as an all‐solid‐state sensor, they provide good physical stability, chemical and thermal resistance, and the ability to be stored dry. This characteristic also makes the sensing element autoclavable or sterilizable with an alkaline solution.^[^ ^6^ ^]^

The remarkable robustness of ISFET sensors with regard to temperature, pressure, and long‐term measurements has made them one of the most common commercially available pH sensors produced by multiple manufacturers worldwide. Nevertheless, to the best of our knowledge, the number of commercial ISFET manufacturers is small, since large sensor companies often do not openly share their ISFET sources.

Our group works on the cultivation and modification of biological organisms for a variety of applications. Our scope are biotechnology and life sciences applications, which are conducted in aqueous media with moderate pH conditions (pH 4 to pH 10) over a runtime of several days. An acceptable level of pH sensing precision is ± 0.1 pH units over the entire duration of the measurements. In some applications, an online measurement with even higher deviations is sufficient if no other sensors are available due to space or dimension restrictions.

Specifically, our intended application is battery‐powered pH measurement over several days in a highly miniaturized, spherical device with a diameter of less than 10 mm and an overall battery capacity of one milliampere‐hour. Therefore, we focused on ISFET manufacturers which offer off‐the‐shelf products that are already within the required range of dimensions for integration into such a system. We purchased and extensively investigated the commercial evaluation products of three companies: Sentron Europe B.V. (NL), Microsens SA (CH), and Winsense Co., Ltd. (TH). Although literature describing the performance of these ISFETs is abundant, studies which compare the characteristics of different commercial products do not exist.^[^ ^7^, ^8^ ^–^ ^9^ ^]^ The exact layer composition, encapsulation materials and manufacturers working point determination is not publicly available. The recommended operating points of the ISFETs, as stated in the respective datasheets, are a constant current demand of 30–100 µA with a working potential of 0.3–0.5 V. The ISFETs power consumption, without further active components, leads to a rather short continuous measurement time.

The known temperature‐I_ds_ dependency^[^ ^10^ ^]^ can be neglected when using ISFETs at their respective recommended working points. These points are calculated from and supported by experimental data provided by the ISFET suppliers. Summarizing the literature results of the isothermal behavior evaluation, if the ISFETs are used outside their respective operating point (i.e., at a higher or lower *I*_ds_ and/or *V*_ds_), the temperature dependency of *V*_gs_ increases. To exclude the influence of isothermal behavior, we kept the temperature constant between calibration and application.

In our study, we demonstrate the reliability and robustness of ISFET sensors from three commercial producers and examine conditioning, long‐term stability, linearity, precision, and response times. Furthermore, we present two strategies to reduce power demand without compromising ISFET performance.

## 2 Experimental Section

### 2.1 Sensors

The ISFETs were purchased from the following companies: Sentron Europe B.V. (Netherlands, pH sensor module), Winsense Co., Ltd. (Thailand, WIPSK‐S), and Microsens SA (Switzerland, MSFET 3330). They were used without modification. According to the datasheets, the operating points are as follows: 1) Sentron: *I*_ds_ 100 µA, *V*_ds_ 0.5 V; 2) Microsens: *I*_ds_ 80–100 µA, *V*_ds_ 0.5 V; and 3) Winsense: *I*_ds_ 30 µA, *V*_ds_ 0.3 V.

The ISFETs were stored dry and used without preparation to evaluate performance. Due to the high cost of the ISFETs (Microsens and Sentron cost between €120 and €180 per sensor, and Winsense costs around €15 per sensor), it is not feasible to use a fresh sensor for each measurement.

The buffer solutions were obtained commercially from VWR (pH 4 ± 0.02: phthalate buffer; pH 6 ± 0.02: citrate buffer; pH 7 ± 0.02: phosphate buffer; pH 8± 0.02: borate buffer; pH 10 ± 0.02: carbonate buffer) and were used directly. The manufacturers recommend using their pH buffer solutions, which contain the same ionic species. However, the potential output linearity of the buffer solutions with different ionic species did not show any effects.

Commercial reference electrodes (RE‐5B from BaSi Inc., US) were used. The stability of the electrodes was tested prior to usage.

### 2.2 Electronics

**Figure 1:**
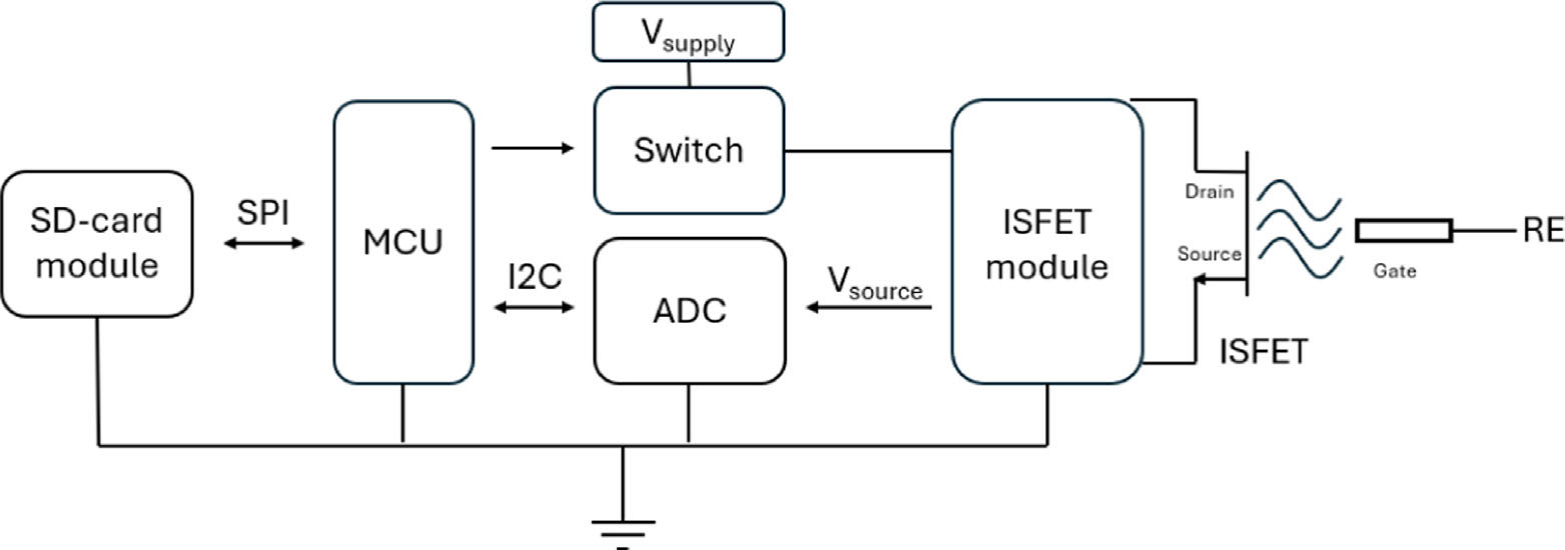
Set‐up for the ISFET evaluation as functional block diagram with SD‐card module, microcontroller, external power supply, switch (LDO or MOSFET), and analog‐digital converter for the recording of the source‐potential of the ISFET. The working point was set by the commercial ISFET modules.

The devices for the ISFET supply (**Figure** **1**, ISFET module) were obtained commercially from Sentron (Sentron Device, SD; an analog front‐end module with a *V*_ds_ of 0.5 V and an *I*_ds_ of 100 µA) and Winsense (Winsense Device, WD; WIPSK‐CB1 with a *V*_ds_ of 0.3 V and an *I*_ds_ of 30 µA).

The microcontroller unit (MCU), which powers the ISFET supply modules and communicates with the analog‐digital converter, is an ESP32 module (Wroom32, AZ delivery, DE). Data was stored externally and analog‐to‐digital conversion was implemented using a 16‐bit AD1115 (Texas Instruments, USA) with a resolution of 62.5 µV at 860 samples per second.

A further LDO (LD2981CM33TR, STMicroelectronics N.V., CH) or a MOSFET (IRF520NPBF, Infineon Technologies AG, DE) was used to control the external power supply of the SD or WD, respectively. The functional block diagram of all units is shown in Figure 1.

### 2.3 Autosampler

**Figure 2:**
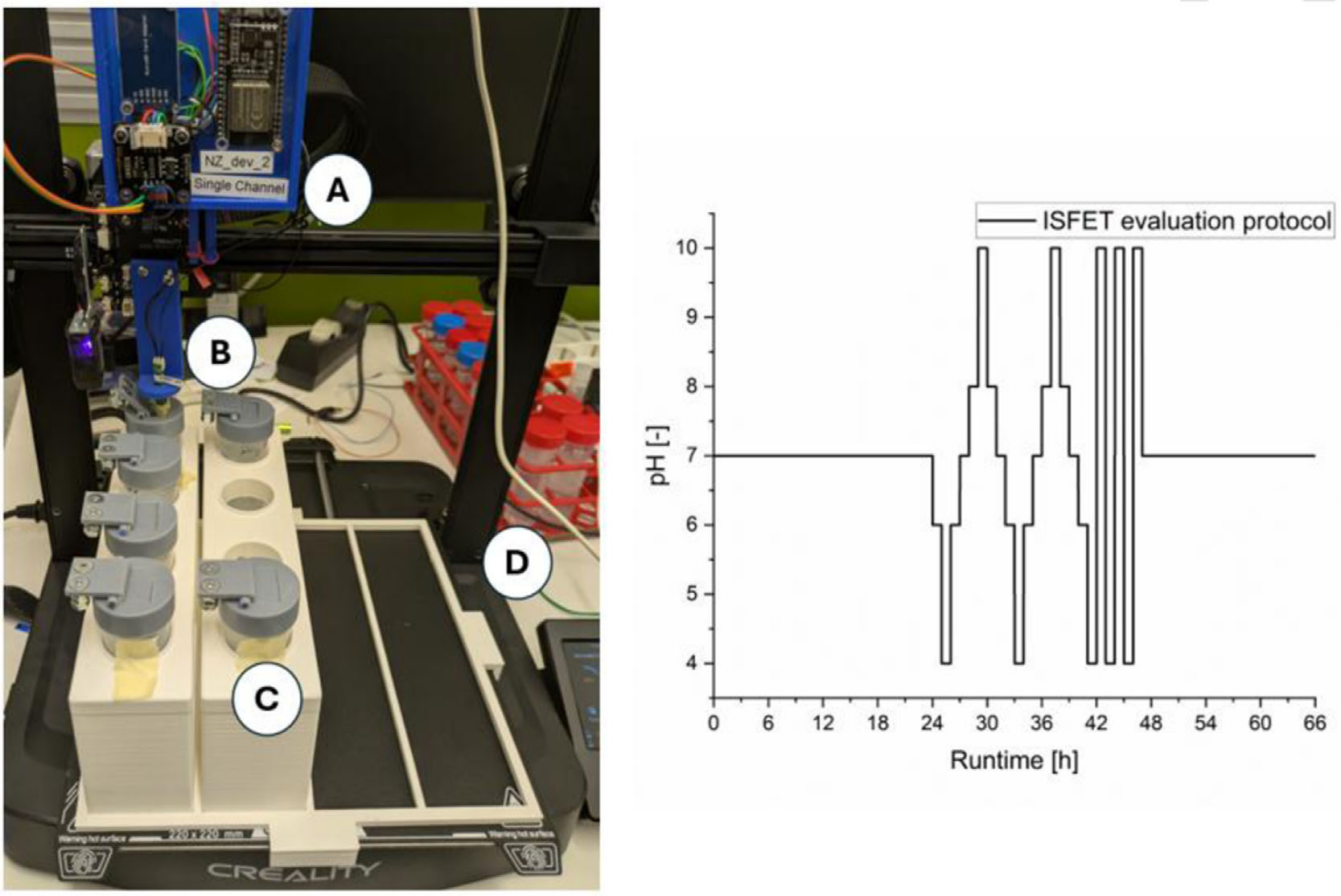
Left: the used autosampler made from a commercial 3D‐filament printer with A: sensor electronics in a sensor holder; B: ISFET and reference electrode; C: sampler holder with caps; D: 3D‐printer. Right: the ISFET long‐term evaluation protocol using a DIY autosampler and five buffer solutions with pH values from 4 to 10 over 66 h.

The long‐term ISFET output stability was evaluated using an automated sampling unit (**Figure** **2**, left). The autosampler consists of a widely used 3D filament printer (Creality Ender‐3 V3SE, firmware version 1.0.6, priced at around €150) that was modified with a modular, 3D‐printed sample holder and a 3D‐printed sensor holder similar to those described in the literature.^[^ ^11^ ^]^ The sample holder was attached directly to the print bed. After removing the hot end, the sensor holder was attached to the assembly using the same screw holes. The initial modification to function as a sampling platform took 1 h, excluding printing time. Resetting the system to a state usable as a 3D printer takes about 30 min. The STL files for the 3D‐printed parts are available in the data repository of the supporting information.^[^ ^12^ ^]^

The sampling method can be adapted to any XYZ movement protocol by directly controlling the machine with G‐code commands. G‐code is highly simplified, and all possible commands are well‐described in the literature (e.g., Marlin Firmware and the G‐code index).^[^ ^13^ ^]^ Each movement can be tested using a direct serial connection to the printer via USB and free open source software such as Pronterface. The method adaptation was done using a simple Excel file with dropdown options for positions, waiting times, and washing steps. This Excel file is included in the supporting information and can be used or adapted for different autosampler applications in a matter of hours.

Another improvement to prevent evaporation was made with a physical barrier that opens and closes with force from above. This modified screw cap is designed for 50 mL reaction tubes from Sarstedt, Corning, and VWR and has been tested.

### 2.4 Proton Activity Dependency (pH)

The evaluation duration and testing protocol were adapted for an application scope of a 66 h measurement time within a pH range of 4–10. Five different pH buffer solutions containing various buffer systems were used to investigate the pH range. The pH change protocol is shown in Figure 2. A washing step was performed between each step, during which the ISFET and the reference were dipped into a water solution for 1 s to avoid carryover. Conditioning, stabilization, and drift evaluation were performed at pH 7 for 24 h prior to the step responses. A qualitative analysis of the 18 h after the step responses at pH 7 shows potential response stability only. A statistical analysis of each ISFET chip, with each method evaluated three times, was not possible due to time restriction. The temperature was kept between 20 and 24 °C due to the environment between day and night. Since there are no potential response changes that correlate with the temperature change in this range for the most prone S5_WD continuous measurement (see Figure S5, Supporting Information) in the 0–24 h and 48–66 h runtimes, we concluded that the temperature influence is negligible.

Linearity was evaluated using the ISFET potential response to pH steps. The precise pH values of the buffer solution were determined using a calibrated commercial pH sensor (WTW, inoLab pH 720, UK) before and after the measurement. These values were then used for the linearity plots. Average ISFET response values for each pH step were determined by selecting a time frame with stable sensor output with a deviation of less than ± 1 mV/10 min. Plotting these values obtained for each pH step between 23 and 49 h of the evaluation protocol results in reliable sensor potential output data for determining linearity.

The precision of the sensors was calculated by determining the standard deviation for each step and using the fit function coming from the linearity fit to obtain the value in pH. The highest deviation is given for each sensor combination in **Table** **1**.

**Table 1:** The obtained ISFET performance values for conditioning time, drifts, linearity fit, precision accuracy, resolution, and response times.

Set	ISFET	Conditioning time [h:m]	Drift [mV day^−1^]	Linearity [mV pH^−1^]	Precision [pH]	Accuracy [R^2^]	T_90_ [s] [pH_4‐>10_]	T_90_ [s] [pH_10‐>4_]	Remarks
Sentron FETs with Sentron Device *continuous*	S2	02:10	+ 0.43 ± 0.01	53.0 ± 0.3	±0.08	0.9992	36 ± 2	37 ± 0	
	S3	02:30	−0.29 ± 0.01	53.4 ± 0.3	±0.09	0.9992	37 ± 1	43 ± 1	
	S4	02:30	−0.33 ± 0.01	53.8 ± 0.7	±0.09	0.9959	34 ± 1	36 ± 1	
Microsens FETs with Sentron Device *continuous*	M2	08:00	−1.35 ± 0.01	57.4 ± 0.2	±0.01	0.9997	38 ± 0	39 ± 1	
	M5	03:30	−1.67 ± 0.01	58.0 ± 0.2	±0.03	0.9996	35 ± 2	37 ± 1	
	M6	06:30	+1.88 ± 0.01	57.4 ± 0.2	±0.03	0.9997	36 ± 1	39 ± 1	
Sentron FETs with Winsense Device *continuous*	S2	00:45	+ 3.79 ± 0.01	50.9 ± 0.4	±0.01	0.9984	28 ± 4	26 ± 2	
	S4	04:30	+ 1.10 ± 0.01	50.0 ± 0.5	±0.01	0.9979	27 ± 11	35 ± 15	
	S5	02:45	+ 1.99 ± 0.01	50.8 ± 0.3	±0.03	0.9990	32 ± 4	31 ± 8	
Microsens FETs with Winsense Device *continuous*	M5	01:00	–	55.7 ± 0.1	±0.01	0.9999	25 ± 1	18 ± 1	two drift dynamics (changes after 13–16 h)
	M6	01:00	–	56.4 ± 0.2	±0.01	0.9998	49 ± 4	49 ± 3	
	M7	01:30	–	56.8 ± 0.2	±0.01	0.9998	74 ± 11	39 ± 1	
Winsense FET with Winsense Device *continuous*	W1	05:00	−30.4 ± 0.05	51.2 ± 1.1	±0.09	0.9890	58 ± 10	38 ± 6	Strong potential noise, shifts and drifts
Sentron FETs with Sentron Device *OnOff*	S2	01:00	+1.57 ± 0.02	51.5 ± 0.5	±0.11	0.9975	40 ± 14 a)	60±0a)	
	S3	01:30	+2.18 ± 0.02	51.5 ± 0.5	±0.02	0.9977	60 ± 0 a)	30 ± 0 a)	
	S4	01:40	+0.56 ± 0.01	48.4 ± 0.7	±0.06	0.9938	40 ± 14 a)	45 ± 15 a)	
Microsens FETs with Sentron Device *OnOff*	M4	01:00	−1.24 ± 0.01	57.6 ± 0.3	±0.09	0.9992	50 ± 14a)	60 ± 0 a)	
	M5	01:00	−0.37 ± 0.02	57.3 ± 0.4	±0.07	0.9988	50 ± 14 a)	60 ± 0 a)	
	M6	00:45	−1.40 ± 0.02	56.2 ± 0.3	±0.03	0.9994	80 ± 28 a)	45 ± 15 a)	
Sentron FETs with Winsense Device *OnOff*	S1	04:00	+8.40 ± 0.03	52.2 ± 0.6	±0.10	0.9971	80 ± 14 a)	75 ± 15 a)	
	S2	04:15	+2.89 ± 0.02	54.0 ± 0.9	±0.08	0.9932	70 ± 14 a)	60 ± 15 a)	
	S4	04:00	+0.95 ± 0.02	49.5 ± 0.7	±0.16	0.9951	60 ± 0 a)	60 ± 0 a)	
Microsens FETs with Winsense Device *OnOff* *66 h*	M4	02:00	+ 6.10 ± 0.17	–	–	–	–	–	Strong potential drift after 16 h
	M5	01:45	−7.41 ± 0.09	–	–	–	–	–	
	M6	02:30	−4.90 ± 0.06	–	–	–	–	–	
Microsens FETs with Winsense Device *OnOff* *24 h*	M1	–	–	57.4 ± 0.2	±0.05	0.9996	60 ± 0 a)	60 ± 0 a)	
	M3	–	–	59.4 ± 0.3	±0.02	0.9994	40 ± 14 a)	45 ± 15 a)	
	M4	–	–	57.1 ± 0.3	±0.07	0.9994	30 ± 0 a)	75 ± 16 a)	
Winsense FET with Winsense Device *OnOff*	W2	–	–	59.7 ± 1.1	±0.12	0.9922	60 ± 0 a)	60 ± 0 a)	Strong potential noise, shifts and drifts

a) Measurement interval of 30 s.

T90 times are obtained as averages from each potential jump response in the time frame between runtime 41—47 h and calculating the 90th percentile of the average ISFET potential response for each step. Our method results in reliable T90 times in measurements with a 1 s interval. For OnOff‐measurements with a 30 s interval, our method yields unreliable T90 times. Therefore, the OnOff‐measurement T90 times are marked with a remark ([a]) in the Data tab. They are used for qualitative analysis only.

### 2.5 Conditioning

The qualitative analysis of the first 12 h of runtime was used to distinguish conditioning and/or soaking from drift behavior. Typically, the initial change in potential ceased, and the potential shifted in the opposite direction. The turning point of the plotted curve was used to estimate the end of the conditioning and/or soaking process.

### 2.6 Drifting Behavior

The time frame of the first 24 h measurement, minus the conditioning time, was chosen. A linear fit was then performed, resulting in a gradient. This effect, which results in this slope, is usually called sensor drift and shows the stability of the sensor's response over time.

### 2.7 Data Handling

The V_gs_ output potentials obtained from the ISFETs were recorded using a widely used 16‐bit analog‐to‐digital converter (ADS1115). The ADS1115 was connected to the MCU via the I2C protocol, and the MCU requested the measured potential at different sample intervals. For continuous measurements, the sampling rate was set to one hertz. For the OnOff‐measurements, the sampling rate was set to one hertz during the Off‐time, and the On‐time was recorded with a 50 Hz sampling rate. All data were written as .txt‐files to an SD card.

The data obtained from all measurements were smoothed using the Savitzky–Golay method with 75 points and second‐order polynomials to exclude clipping data points recorded while switching samples. For the OnOff‐measurements an additional switch setting value was used. During postprocessing the data from the OnOff‐measurments, a Python script averaged the recorded 50 data points before Off‐switching to a single value for each On‐phase and excluded all other data points (i.e., the Off‐phases) from the result. The raw data for each experiment can be obtained from the data repository.^[^ ^12^ ^]^

To compare the potential output of different offset‐shifted ISFETs, the potential at 24 h of runtime was set to zero volts.

The ISFETs are grouped into sets by manufacturer and working conditions (see Table 1). Due to the large number of measurements for each set of three with different settings and manufacturers, the figures for each set are shown in the Supporting Information. To compare each set of three with different manufacturers and operating conditions, an exemplary sensor potential output was chosen and is presented in this work. OriginPro 2024b was used for the data analysis.

### 2.8 Power Consumption

The power consumption of the ISFETs was calculated using the constant working point only (*I*_ds_ and *V*_ds_) resulting in 50 µW (Sentron device) and 9 µW (Winsense device). By turning the device On and Off, a reduction of these values by the fraction of the On‐time was calculated.

In this article, we neglected power consumption by the active components of the device CVCC‐circuits from the manufacturer. Both used circuits (SD and WD) contain further active ICs for either connectivity, further temperature measurement, or battery status representation by LEDs and therefore would not be comparable.

## 3 Results

The commercially obtained ISFETs (S1‐S5 from Sentron, M1‐M7 from Microsens, and W1‐W2 from Winsense) were tested with the recommended working points set by the Sentron (SD) or Winsense (WD) commercial measurement devices for their conditioning time and long‐term performance. Due to the amount of data, only one example is shown for each commercial manufacturer's ISFETs’ potential output over 66 h of runtime. The resulting linear pH response is derived from the averaged potential step responses, as described in the Experimental Section.

After fully evaluating the properties of the sensors at their respective working points, the Sentron and Microsens sensors were evaluated at a decreased working point set by WD. Finally, an OnOff‐strategy that reduced the on‐time of the ISFETs by 93% was established, and the ISFETs were tested for long‐term performance with this strategy, both at the recommended and at a decreased working point. The conditioning, drift rate, linearity, precision, accuracy, and T90 response times of all the investigated ISFETs and all the power‐reducing strategies are presented in Table 1.

### 3.1 Long‐Term Performance at Recommended Working Point

**Figure 3:**
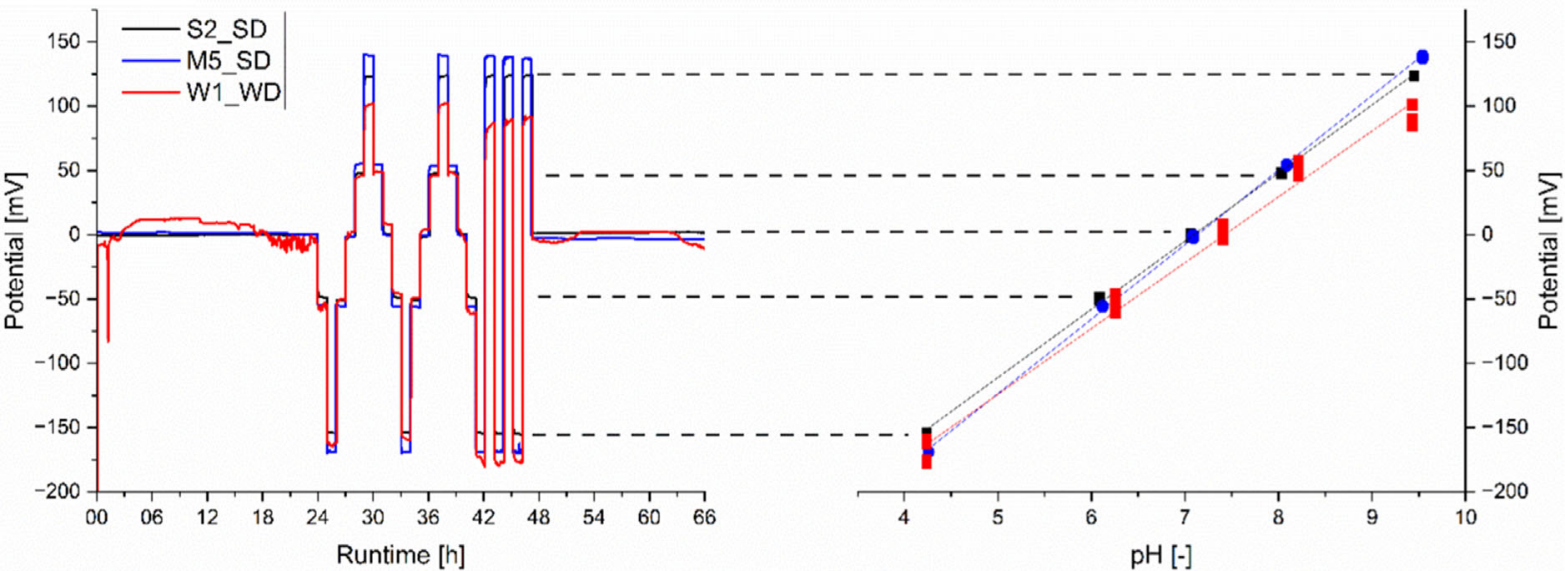
Left: potential response of ISFETs S2, M5 measured by the SD, and W1 by the WD over a runtime of 66 h in five different pH buffer solutions ranging from pH 4 to pH 10. Right: the corresponding linear fit of the averaged potential response values for each pH buffer solution.

All of the ISFETs (S2, S3, and S4; M2, M5, and M6; and W1) and their respective sensing devices (SD and WD) were used without conditioning during the 66 h evaluation process. This process used a DIY autosampler and buffer solutions with different pH values. The results are shown in Table 1. **Figure** **3** shows one measurement with an ISFET from each manufacturer at its recommended working point.

Manufacturers propose different initial soaking and/or stabilization times for their products. In this work, we test for initial drifting, or conditioning, by measuring the potential output of the ISFETs in a pH 7 buffer solution under constant environmental conditions for 24 h. The conditioning times are listed in Table 1.

Sentron ISFETs (S2, S3, and S4) demonstrate stable potential output in less than 2.5 h. The Microsens ISFETs (M2, M5, and M6) require a maximum of 8.0 h of conditioning. A drift of −1.67 to + 1.88 mV day^−1^ was obtained. The Winsense ISFET (W1) exhibits random drifting until the pH buffer solution changes after 24 h. Of the seven ISFETs tested, all showed a pH‐dependent linear response with slope ranges clustered by manufacturer: Sentron (53.0–53.8 mV pH^−1^), Microsens (54.7–58.0 mV pH^−1^), and Winsense (51.2 mV pH^−1^). The Microsens and Sentron ISFET sets are similar in accuracy and stability, and both result in higher accuracy than the Winsense ISFET. In our evaluation set‐up, the precision of the Sentron devices is lower with ±0.08−0.09 pH, than Microsens with ±0.01−0.03 pH. T90 response times do not follow a trend among the different sets and range from 34 to 58 s.

### 3.2 Long‐Term Performance at Reduced Working Point

**Figure 4:**
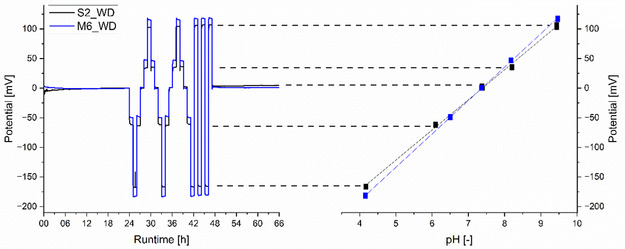
Left: potential response of ISFETs S2 and M6 measured by the WD over a runtime of 66 h in five different pH buffer solutions ranging from pH 4 to pH 10. Right: the corresponding linear fit of the averaged potential response values for each pH buffer solution.

ISFETs S2, S4, and S5 and M5, M6, and M7 were tested at a decreased working point set by the WD, to evaluate the strategy of reducing sensor power demand by changing the field effect transistor's potential and constant current. The 66 h evaluation process was used again, as shown in **Figure** **4** for one example from each set.

The Sentron ISFET set (S2, S4, and S5) with WD shows stable potential output after conditioning for 45–270 min, with a drift of 1.10–3.79 mV day^−1^. The Microsens ISFET set (M5‐M7) with WD was conditioned for a maximum of 90 min. This was followed by drift behavior with two dynamics and a change point between 13 and 16 h (see Figure S10, Supporting Information, for details). All ISFETs demonstrate a pH‐dependent linear response, with Sentron showing a slope range of 50.0–50.9 mV pH^−1^ and Microsens showing a slope range of 55.7–56.8 mV pH^−1^. Microsens ISFETs demonstrate greater accuracy and stability than Sentron sensors and the precision is for both manufacturers between ± 0.01−0.03 pH. The T90 response times do not follow a trend among the different sets and range from 26 to 90 s.

### 3.3 OnOff‐Strategy

**Figure 5:**
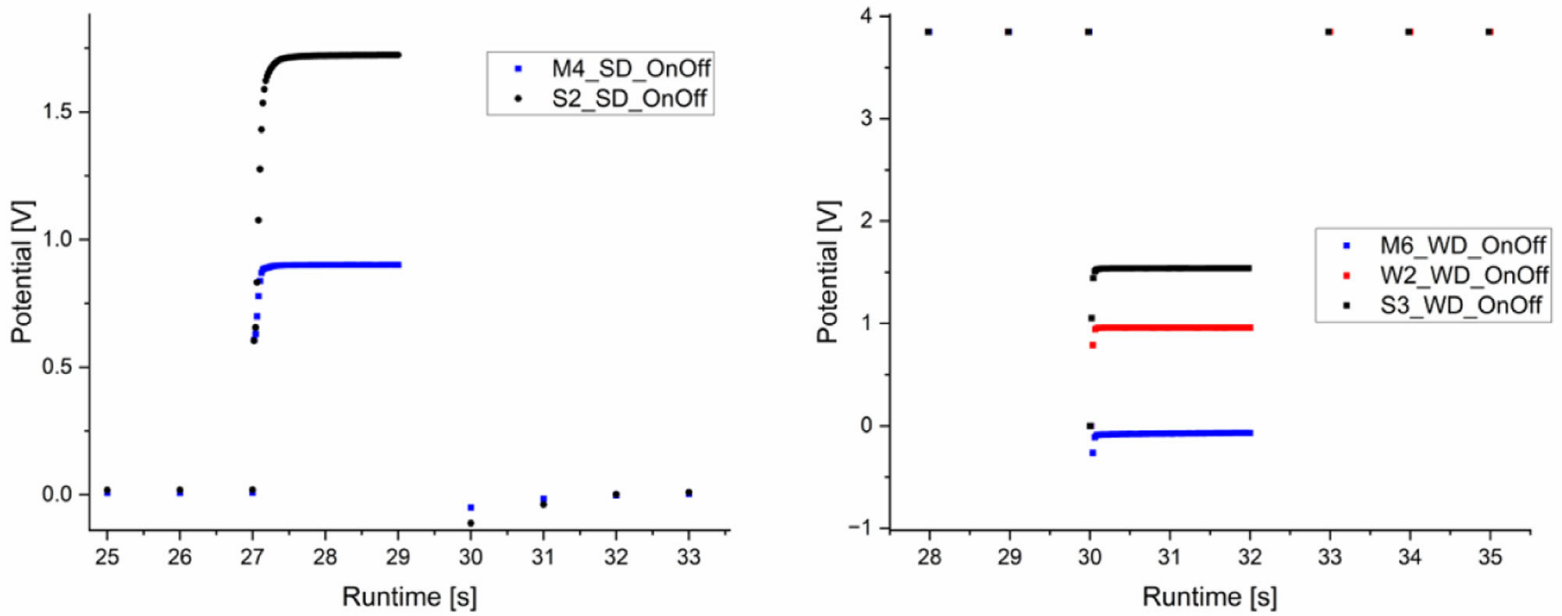
Left: potential response of one interval OnOff‐measurement with an example of each ISFETs for SD. Right: potential response of one interval OnOff‐measurement with an example of each ISFETs for WD.

To further reduce power consumption, an MCU‐controllable switch was used to turn the SD and WD measurement devices On or Off. A sampling rate of 50 Hz was used in the On‐state to evaluate dynamics. The process was controlled and recorded by DIY electronics. **Figure** **5** shows one random example of the dynamics until a stable potential output was reached using different manufacturers’ ISFETs and the two devices, SD and WD.

Figure 5 shows the dynamic of the ISFET On‐switching for S2 and M4 with the SD. Both ISFETs reach a stable output within 2 s of the On‐state. S2 had a longer ramp‐up phase until reaching a potential plateau. Quantification is not useful since temperature, pH value, and electrical measurement sensor noise are not comparable. Using the SD, the ISFETs demonstrate stable potential output in less than 1 s. Therefore, to take an average over 1 s per measurement cycle, a total On‐state of 2 s was chosen.

On the right side, Figure 5 shows the dynamics of the ISFET On‐switching for S3, M6, and W2 with WD. The sampling rate in the On‐state is 50 Hz again, and an On‐state time of 2 s was chosen since initial testing showed a stable potential output after 2 s. W2 and S3 reach a stable potential output within 1 s of the On‐state. M6 shows a potential increase even after 2 s. However, to keep the results comparable for all ISFETs, the OnOff‐protocol was set to 2 s of On‐state and 28 s of Off‐state for all subsequent long‐term measurements.

### 3.4 OnOff, Long‐Term Performance at Recommended Working Point

**Figure 6:**
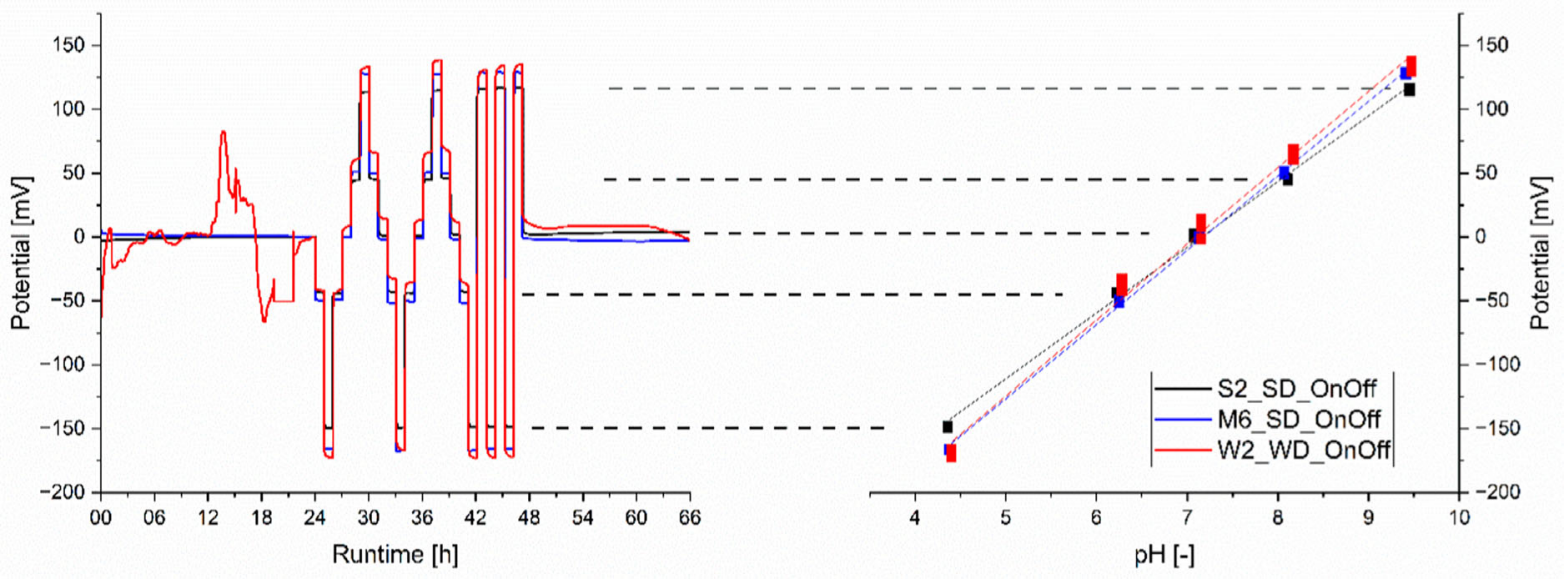
Left: potential response of ISFETs S2 and M6 measured by the SD and W2 by the WD with the OnOff‐protocol over a runtime of 66 h in five different pH buffer solutions ranging from pH 4 to pH 10. Right: the corresponding linear fit of the averaged potential response values for each pH buffer solution.

To evaluate the OnOff‐strategy, we first tested the ISFETs at their recommended operating points, turning them Off for 28 s (sampling rate of 1 Hz) and On for 2 s (sampling rate of 50 Hz). As described in the data handling mode, a single potential value was averaged for each 30 s interval, and the switched ISFET was evaluated using the 66 h evaluation method. **Figure** **6** shows an exemplary potential output for each ISFET set.

The ISFETs from Sentron (S2, S3, and S4) and Microsens (M4, M5, and M6) all show stable outputs after less than 2 h of conditioning. The Sentron set shows a drift rate of + 0.56 to + 2.18 mV day^−1^. The Microsens showed negative potential drifts from −0.37 to −1.40 mV day^−1^. However, the Winsense W2 ISFET shows random potential output drifts and shifts before the first buffer solution change after 24 h of runtime. All seven tested ISFETs on SD and WD show linear pH‐dependent potential output. The Sentron ISFETs have the lowest slope range, 48.5–51.5 mV pH^−1^; the Microsens ISFETs have a steeper slope range of 56.2–57.6 mV pH^−1^; and the Winsense W2 ISFET has the highest slope range at 59.7 mV pH^−1^. The ISFETs’ accuracy and stability are in the order of Sentron, followed by Microsens and Winsense. The precision shows a broad range between the single ISFETs in each set for both Microsens (±0.03−0.09 pH) and Sentron (± 0.02−0.11 pH). T90 response times do not show any trends with respect to the sets and range from 45 to 80 s.

### 3.5 OnOff, Long‐Term Performance at Reduced Working Point

**Figure 7:**
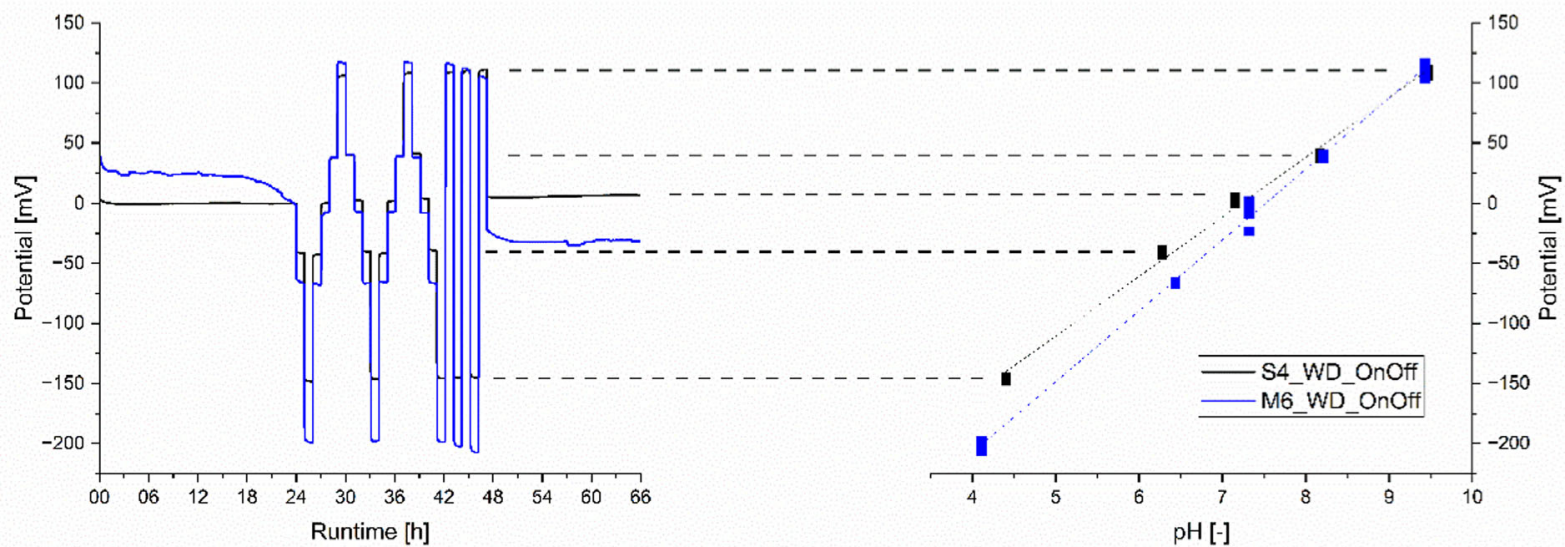
Left: potential response of ISFETs S4 and M6 measured by the WD with the OnOff‐protocol over a runtime of 66 h in five different pH buffer solutions ranging from pH 4 to pH 10. Right: the corresponding linear fit of the averaged potential response values for each pH buffer solution.

Finally, the two power‐reducing strategies were combined. ISFETs S1, S2, and S4, as well as M1, M3, M4‐M6, were tested with the reduced working point using the OnOff‐protocol with WD. **Figure** **7** shows one example of each set.

All three Sentron ISFETs (S1, S2, and S4) show stable outputs after around 4 h, with drift rates ranging from 0.95 to 8.40 mV day^−1^. These sensors also demonstrate a pH‐dependent linear potential response over 66 h. The slope ranges from 49.5 to 54.0 mV pH^−1^ with high accuracy and a reduced precision of ±0.08−0.16 pH. The T90 values are in the comparable range of 60–80 s. The ISFETs from Microsens (M4, M5, and M6) show stable outputs after less than 2.5 h of conditioning. They then exhibit drift rates ranging from −7.41 to + 6.10 mV day^−1^. After around 16 h at pH 7, all ISFETs show a loss of stability, followed by a sigmoidal potential drift for the next 50 h. This set of ISFETs could not be used for linear response analysis. Therefore, a second set of Microsens ISFETs (M1, M3, and M4) was tested for linearity and potential step response directly, without the 24 h preconditioning protocol with WD and the OnOff‐protocol. All three sensors showed a linear, pH‐dependent potential response between 57.1 and 59.4 mV pH^−1^ with high accuracy and comparable T90 values between 30 and 75 s.

Further evaluation of the unexpected potential drifts after 16 h in OnOff‐mode without a pH change was not performed.

## 4 Discussion

The evaluation method using the 3D‐printed autosampler proved reliable for evaluating the ISFETs. Further alterations involving different pH buffer solutions, temperature settings, and UV light intensities were not evaluated. The known temperature dependency of the literature^[^ ^10^ ^]^ was thereby neglected and kept fairly constant in our experiments with a temperature range of 20–24 °C. For our intended long‐term measurement of a turbulent biological system, the determined sensor specifications and performance are acceptable.

Of the 14 ISFETs tested from three manufacturers (Sentron, Microsens, and Winsense), all showed a linear, pH‐dependent output when used with two devices: SD (*I*_ds_ = 100 µA, *V*_ds_ = 0.5 V) and WD (*I*_ds_ = 30 µA, *V*_ds_ = 0.3 V). The ISFETs were used for several evaluation methods and showed stable outputs until an abrupt loss of function occurred for several sensors. Since the ISFETs were designed differently (Sentron with epoxy resin encapsulation on a flex connector, Microsens with epoxy resin encapsulation on a rigid PCB with a connector, and Winsense with epoxy resin encapsulation attached to jumper wires), the reasons for the loss of function could not be systematically compared. Of the nine Winsense ISFETs tested, a first batch of four sensors obtained commercially failed within the first hours of usage, while a second batch of five sensors had four functioning and one nonfunctioning sensor. All ISFETs obtained from the other manufacturers worked initially. Therefore, the Winsense statistical evaluation of at least three ISFETs was shortened to a single exemplary one.

With the test setup used and the buffer solutions of different ionic strengths and types of ions, all ISFETs worked linearly with precision of ± 0.1 pH or better when the manufacturer‐advised ISFET working points (*I*_ds_ and *V*_ds_) and continuous measurement were used. Response times are less than 60 s for a change from pH 4 to pH 10 and vice versa for an unstirred setup at room temperature and moderate ionic strength. However, a comparison with the manufacturer's datasheet is not possible due to changes in handling and application outside the manufacturer's recommendations.

Even when obtained from different batches and evaluated at different sensor lifetimes, sets of three ISFETs from each manufacturer and measurement mode exhibited systematic differences compared to others. First, the ISFETs were measured continuously at their respective working points, followed by a reduced working point.

The Sentron ISFETs (S2‐S5) showed comparable conditioning times at the recommended working point with SD and at a decreased working point with WD. The linear pH‐dependent potential response was lowest among the different manufacturers (SD: 53.0–53.8 mV pH^−1^ and WD: 50.0–50.9 mV pH^−1^), with high accuracy. The precision for the Sentron ISFETs together with their recommended electronics SD is comparably low with ± 0.08−0.09 pH. For the reduced working point, the precision improves to ± 0.01−0.03 pH. For our scope in biotechnology, a precision of less than ±0.1 pH is sufficient. Response times were comparable (SD: 36–43s and WD: 27–35 s), but difficult to interpret for a non‐stirred system.

Microsens ISFETs showed comparably long conditioning times at the recommended working point with SD during continuous measurements. With WD, a qualitative conditioning time of 60–90 min was followed by a change in linear drift dynamics after 13–16 h at a constant pH of 7. The slopes are steeper with higher values for SD (57.4–58.0 mV pH^−1^), compared to WD (55.7–56.8 mV pH^−1^). Both have very high accuracy and the obtained precision values are almost perfect with ±0.01−0.03 pH. Response times differ greatly using WD (18–90 s), compared to SD (35–39 s).

Continuous measurement with the Winsense ISFET (W1) at the recommended working point with WD showed fair conditioning time, but an erratic drift behavior at a constant pH of 7. The slope was lowest for the ISFETs at their respective working points, at 51.2 mV pH^−1^, with high deviation and low accuracy. The precision of ± 0.09 pH is fair. The response times were comparable to those of the other ISFETs at 38–58 s.

Although a calculated decrease in power demand of 82%, from 50 *µW* (SD) to 9 µW (WD), was already achieved, OnOff‐measurements with a much lower power demand were evaluated. Initial tests showed stable potential output for all three manufacturers’ ISFETs with 28 s in the Off‐state and 2 s in the On‐state. The 66 h evaluation protocol was used again.

The set of Sentron ISFETs (S2‐S4) in OnOff‐mode with SD showed a shortened conditioning time of 60–100 min and a higher drift rate of 0.56–2.18 mV day^−1^ after conditioning. There was no correlation between the conditioning times and the drift behavior. Compared to continuous measurements, the potential response slopes decreased to 48.4–51.5 mV pH^−1^, with reduced accuracy and precision of ± 0.02–0.11 pH. Due to the long sampling interval, the response times were only qualitatively evaluated as described in the methods.

The set of ISFETs purchased from Microsens (M4‐M6) and used in OnOff‐mode with SD also showed a shortened conditioning time of 45–60 min. The drift rate of −0.37 to −1.40 mV day^−1^ is in the same range as the continuous measurements. However, a slightly lower slope of 56.2–57.6 mV pH^−1^ was observed, together with reduced accuracy and reduced precision of ±0.03−0.09 pH. Response times were longer than in continuous mode.

An exemplary Winsense ISFET (W2) in the OnOff‐mode at the recommended working point with WD exhibited significant shifts and drifts in the output before the first change of the pH buffer solution. This behavior was observed with several Winsense ISFETs (not shown in this work). The pH‐dependent linearity exhibited a high slope of 59.7 mV pH^−1^ and fair accuracy, with a precision of ± 0.12 pH. The response times are comparable to those in continuous mode. For our application, the performance of Winsense’ ISFETs does not meet our requirements.

Finally, a combination of sensor power demand reduction strategies was used for the Sentron and Microsens ISFETs with WD. Sets of three were investigated in the 66 h evaluation protocol.

Microsens sensors did not demonstrate a potential sensor output for measurements exceeding 24 h with a reduced working point in OnOff‐mode. Therefore, two sets with different evaluation protocols were used to compare a) conditioning and drift and b) linearity, accuracy, precision, and response times, respectively. To implement both power‐reducing strategies, the application runtime should be shorter than a day. The set that was measured for 24 h with reduced working point (WD) and OnOff‐mode showed a high pH‐dependent potential slope of 57.1–59.4 mV pH^−1^, with high precision (0.02–0.07 pH) and accuracy, and comparable response times of around 60 s. The set measured for 66 h showed a conditioning time range between 1.75 and 2.50 h with the following drifts between −7.41 and + 6.10 mV day^−1^.

The Sentron ISFET set (S1, S2, and S4) with WD in OnOff‐mode showed an increased conditioning time of around 4 h, followed by a broad range of drift rates between 0.95 and 8.40 mV day^−1^. The pH‐dependent potential response slopes were higher (49.5–54.0 mV pH^−1^) than those in OnOff‐mode at the recommended working point, together with reduced accuracy and longer response times. Overall, this performance is acceptable for many applications in which the geometry or dimensions of the analyte of interest must be measured by a battery‐powered system. It provides stable output precision of ± 0.08–0.16 pH over 66 h of measurement. In continuous measurement mode, the overall power demand decreased from 50 to 9 µW by selecting the lower working point, which is described in more detail in 2.8. The OnOff‐measurement further decreased the field effect transistor power demand by 93.3%. Combining both strategies led to a calculated decrease in power demand of 98.8% for the ISFET sensors.

## 5 Conclusion

This work investigated the potential application of pH sensors based on ISFET technology in biotechnology, focusing on their long lifetime and application scope in continuous measurements. This works goal was evaluating ISFET sensors and not developing the read‐out circuitry. The measurement electronics were obtained commercially from Sentron and Winsense. We developed evaluation protocols for typical biotechnology applications involving an aqueous medium of interest with a pH range between 4 and 10 and a runtime of several days in a temperature‐controlled environment. We also developed a low‐cost, 3D‐printed autosampler with direct G‐code control for sensor evaluation and obtained commercially available ISFET sensor batches from three different manufacturers: Sentron, Microsens, and Winsense. To the best of our knowledge, a direct comparison of these commercially available ISFETs has not been published. In this study, we demonstrated the reliability of this sensor type and its performance limits under nonideal preparation and operating conditions. Since we intend to incorporate pH sensors into battery‐powered applications with low power storage capacity, we aimed to reduce the sensors’ power consumption. We evaluated two strategies: a) reducing power consumption by shifting the working point (*I*_ds_ and *V*_ds_), and b) implemented an OnOff‐strategy of reducing the sensor's duty cycle to achieve an acceptable measurement interval of 30 s. Finally, we combined both strategies to lower power consumption even further to a calculated consumption decrease of 98.8%.

We found that both Microsens and Sentron offer ISFETs with reproducible, high‐performance sensor output at their recommended working points for continuous and OnOff‐measurements. All ISFETs showed limitations in their overall performance with best performers being 1) Winsense, with the highest slope of 59.7 mV pH^−1^; 2) Microsens, with the highest potential output precision under recommended working point conditions (± 0.01−0.03 pH) with SD and reduced working conditions (± 0.01 pH) with WD; and 3) Sentron, with the highest stability under both power‐reducing strategies.

In future work, we will design a highly miniaturized, self‐made readout circuit and explore the combination of the developed strategies with commercial ISFETs and micro‐reference electrodes.

## Supporting Information

The supporting information with further details about the autosampler, a G‐code creator and figures for all measurements are provided.

## Conflict of Interest

The authors declare no conflict of interest.

## Supporting information

Supplementary Material

## Data Availability

The data that support the findings of this study are openly available in [Center for Open Science, Inc.] at [https://doi.org/10.17605/OSF.IO/UFEA6], reference number [12].
