# Supplementary material for: The Performance of Commercial pH‐Sensitive Ion‐Selective Field Effect Transistors
Source: ChemistryOpen. 2025 Sep 24;14(12):e202500361. doi: 10.1002/open.202500361 (PMC12680566; doi:10.1002/open.202500361)
Supplement: Supplementary file 1 — Supplementary Material [file OPEN-14-e202500361-s001.pdf]

TECHNISCHE UNIVERSITÄT DRESDEN

int

Faculty of Mechanical Science and Engineering Institute of Natural Materials Technology

Chair of Bioprocess Engineering

Supporting Information:

The Performance of Commercial pH-Sensitive Ion-Selective Field Effect Transistors (ISFETs)

Ziebart, Nandor; Gießel, Alexander; Walther, Thomas

Technische Universität Dresden, ZINT Campus, 01069 Dresden, Germany

Mail to: nandor.ziebart@tu-dresden.de

Contents

Autosampler 1

How to use the Excel-GCode creator 2

ISFET sensor potential output 3

Sentron FETs with Sentron Device *continuous* 4

Microsens FETs with Sentron Device *continuous* 5

Sentron FETs with Winsense Device *continuous* 6

Sentron ISFET S5 with Winsense Device *continuous* with temperature 7

Microsens FETs with Winsense Device continuous 8

Winsense FETs with Winsense Device *continuous* 9

Sentron FETs with Sentron Device *OnOff* 10

Microsens FETs with Sentron Device *OnOff* 11

Sentron FETs with Winsense Device *OnOff* 12

Microsens FETs with Winsense Device *OnOff 66 h* 13

Microsens FETs with Winsense Device *OnOff 24 h* 14

Winsense FETs with Winsense Device OnOff 15

Autosampler

The used autosampler is a low-cost solution made from a commercial 3D-filament printer, a widely used Creality V3SE. If purchased, the starting test run of the printer needs to be performed before disassembly for autosampling. In some instances, the printer

firmware froze in the initializing process. Creality offers the firmware together with a how-to for setting up external data storage for booting.

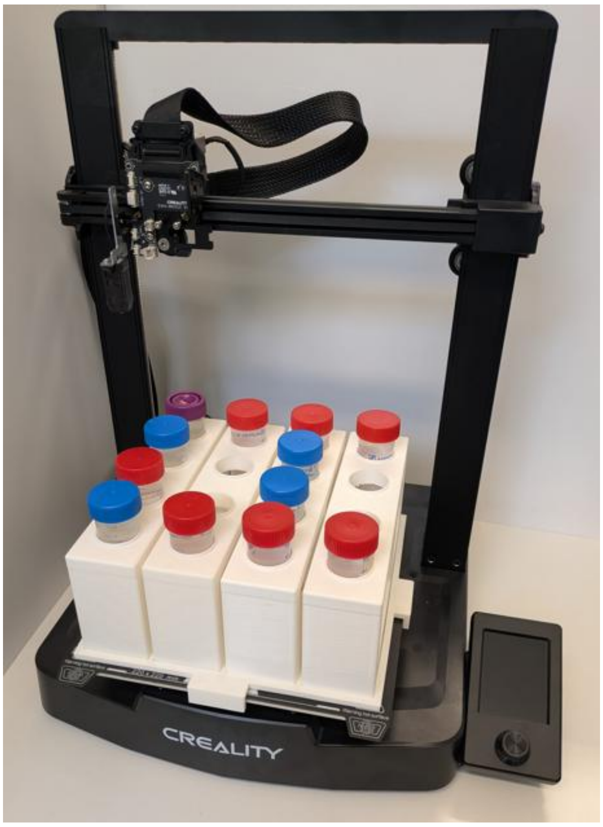

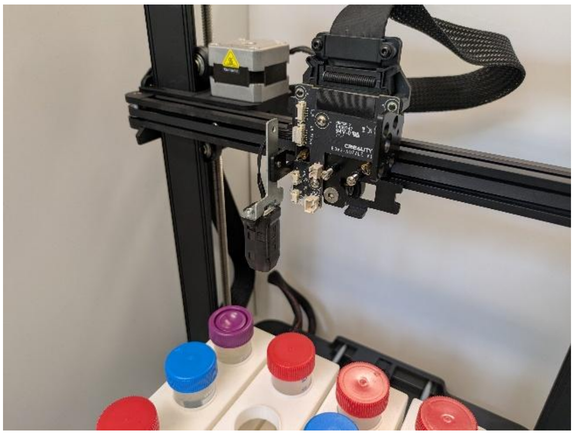

Figure 1: Left: autosampler with sample holder; right: close-up of the detached nozzle-unit.

Teaching and testing the positioning of the xyz-sampler can be done using Pronterface. Choosing the correct COM-port and a baud rate of 115200 works with the chosen printer. Beware that after starting the printer, you must run the auto-homing function. Therefore, a quick attachment/detachment solution for every component which is lower than the z-axis distance sensor AND everything attached to the hot bed is necessary.

An Excel-file for a working GCode-Creator together with the printing files of the sample holder is part of the paper attachment.

How to use the Excel-GCode creator

1. The positioning and numbering of the two trays are illustrated on the left in the main sheet. Below are the exact positions for fine tuning (using Pronterface).

2. The initialization of the printer and setting of some machine code properties are done in the upper right corner. Same for the flushing procedure of the sensor.
3. Marked in red is the necessary information you need to provide. The drop-down menu is based on the second sheet (Positions). Fine tuning for the positions can be set there.
4. Marked in green, you can use the drop-down menu to set the positions you want in any order and the time in seconds, the sampler should stay there. Each setting will fill automatically the column (Q) with the respective GCode. The machines need a line break after each command. Don't be confused, the excel-cell only shows the first line of the full command.
5. Cell H37 shows the fully expanded GCode for the printer, which can be transferred to the printer by copying L37 to a text editor, removing the quotation marks in the beginning and the end of the code and save it as ".gcode" to the external storage (SD-card) for the printer.

Without knowing the printer firmware in detail, there will be no safety sensors. Be careful and double check the positions with the adjustment of glassware and expensive materials. Stay safe and have fun.

ISFET sensor potential output

The commercial ISFETs are used without any preparation in five pH buffer solutions with different pH values. All raw data was smoothed (Savitzky Golay 75 points, second polynomial order) for an exclusion of recorded data-points outside of buffer solutions, when the autosampler was moving. All ISFETs show different potential Offsets – for comparison, the ISFET potential response of the timepoint before the first change of buffer solution was chosen as setpoint to zero volts.

Sentron FETs with Sentron Device *continuous*

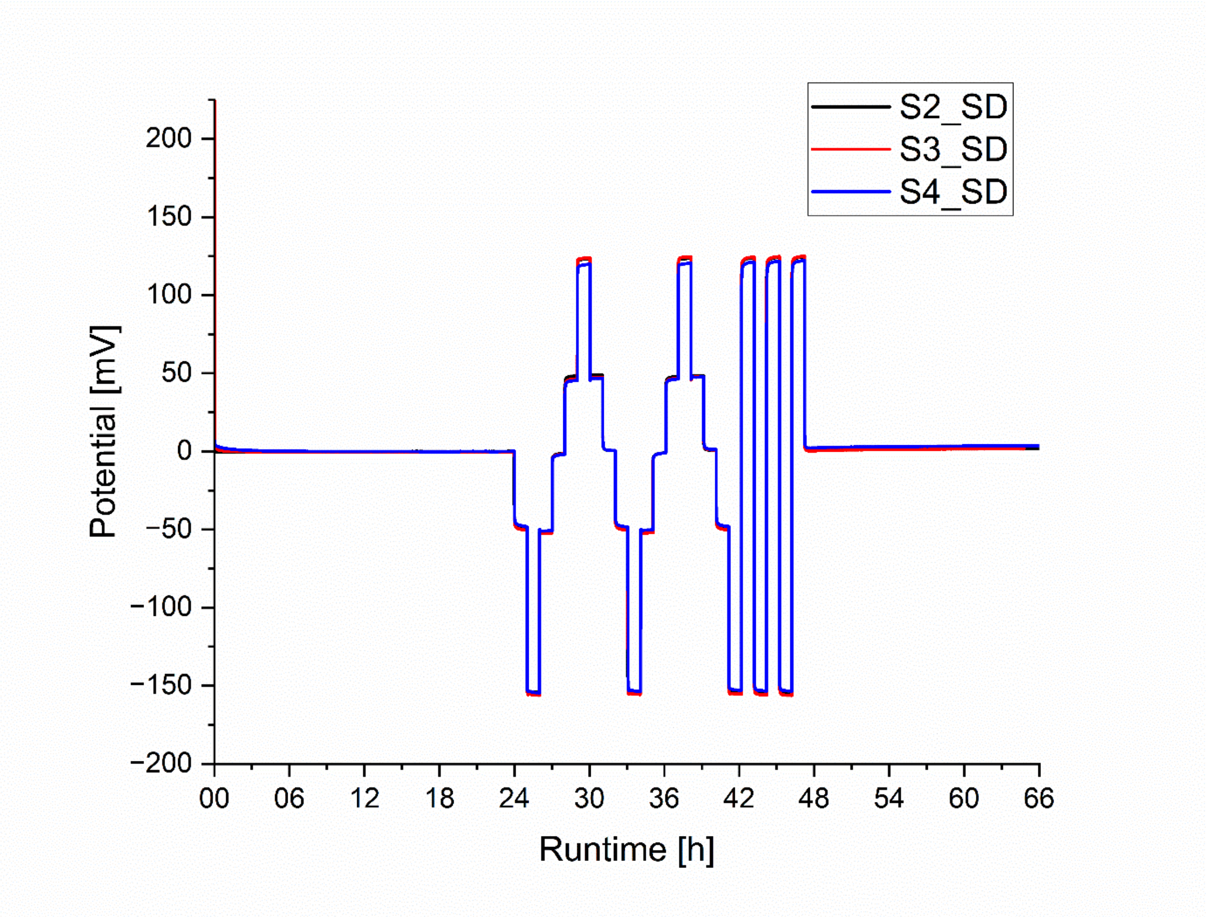

Figure 2: The stacked ISFET potential response to the 66 hours evaluation protocol with the self-made autosampler of S2, S3 and S4 with SD in continuous measurement.

Microsens FETs with Sentron Device *continuous*

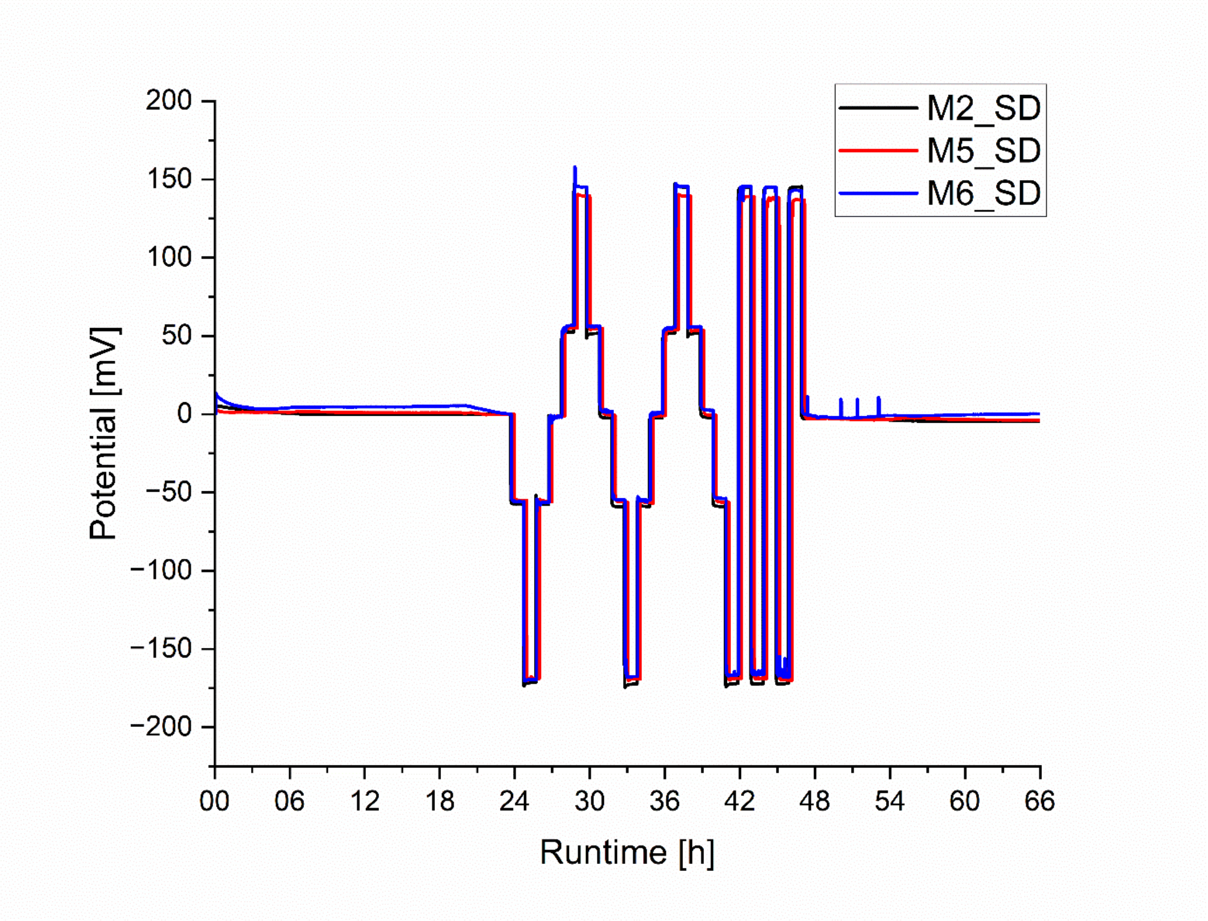

Figure 3: The stacked ISFET potential response to the 66 hours evaluation protocol with the self-made autosampler of M2, M5 and M6 with SD in continuous measurement.

Sentron FETs with Winsense Device *continuous*

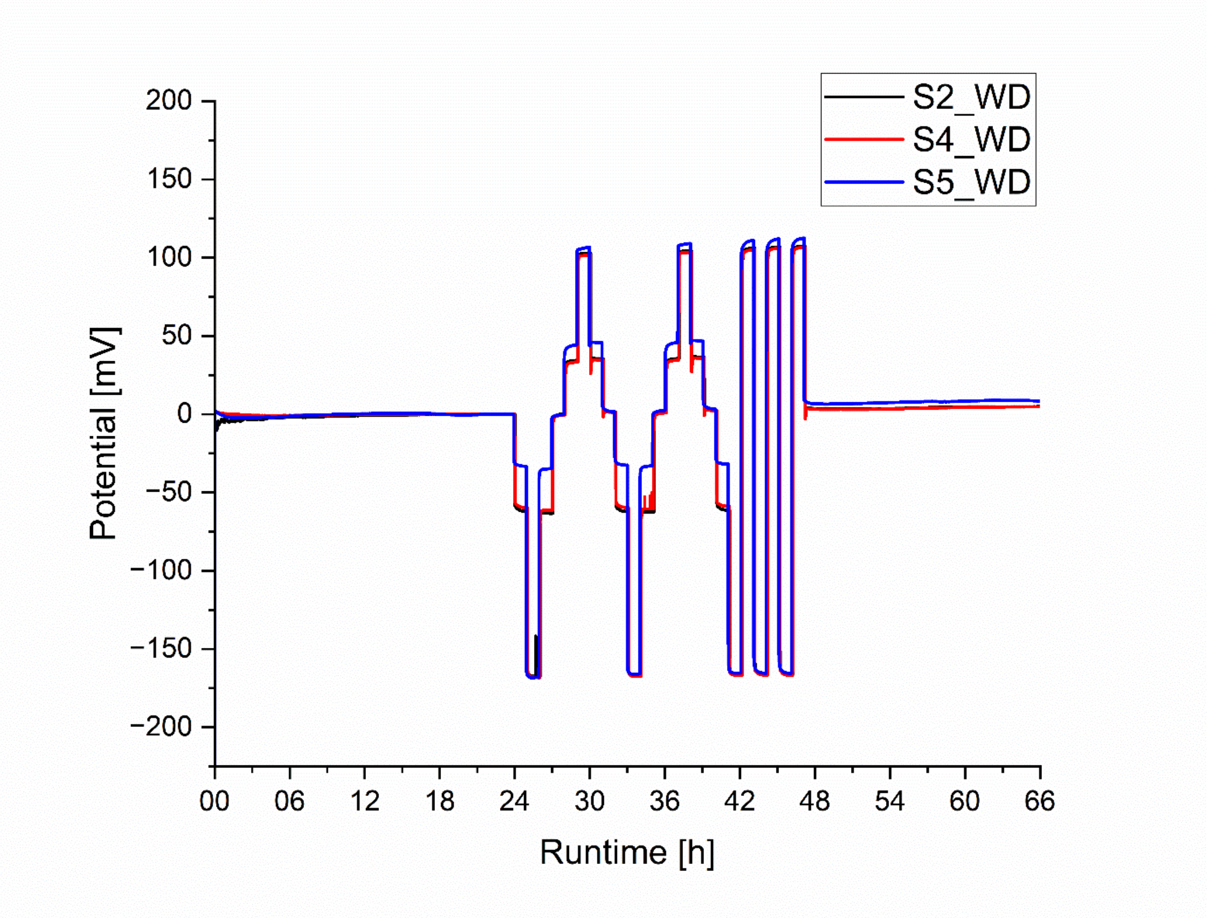

Figure 4: The stacked ISFET potential response to the 66 hours evaluation protocol with the self-made autosampler of S2, S4 and S5 with WD in continuous measurement.

Sentron ISFET S5 with Winsense Device *continuous* with temperature

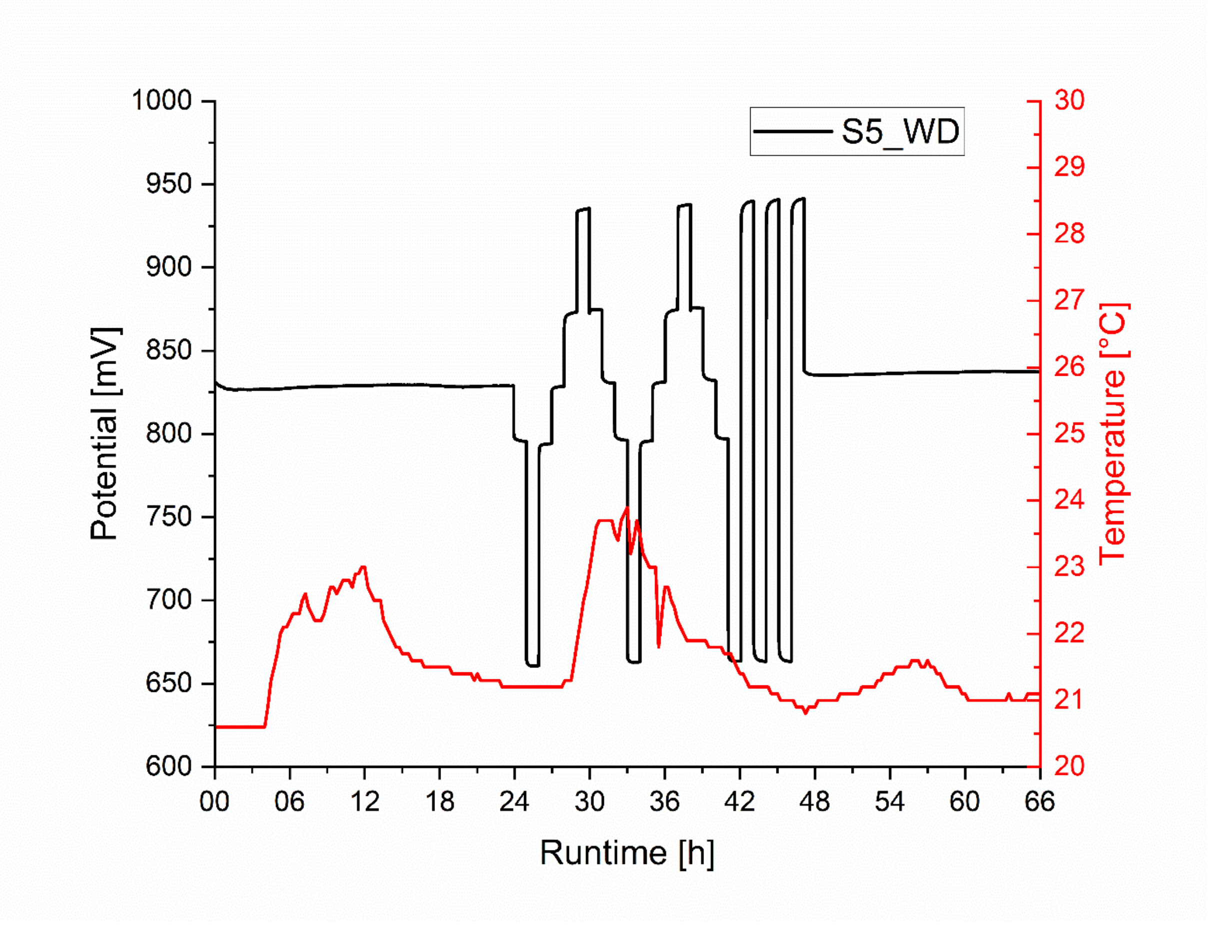

Figure 5: ISFET potential response to the 66 hours evaluation protocol of S5 with WD in continuous measurement stacked with the environmental temperature.

Microsens FETs with Winsense Device continuous

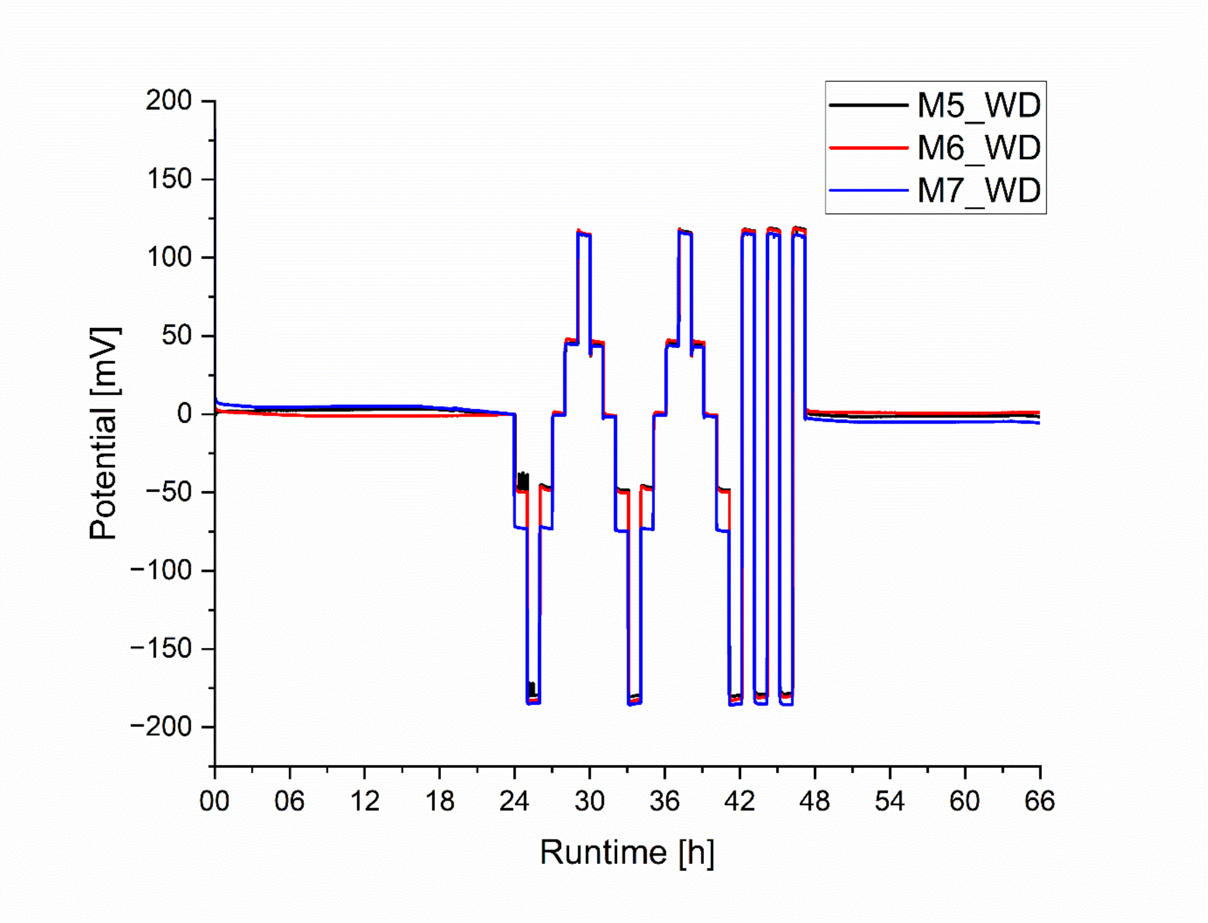

Figure 6: The stacked ISFET potential response to the 66 hours evaluation protocol with the self-made autosampler of M5, M6 and M7 with WD in continuous measurement.

Winsense FETs with Winsense Device *continuous*

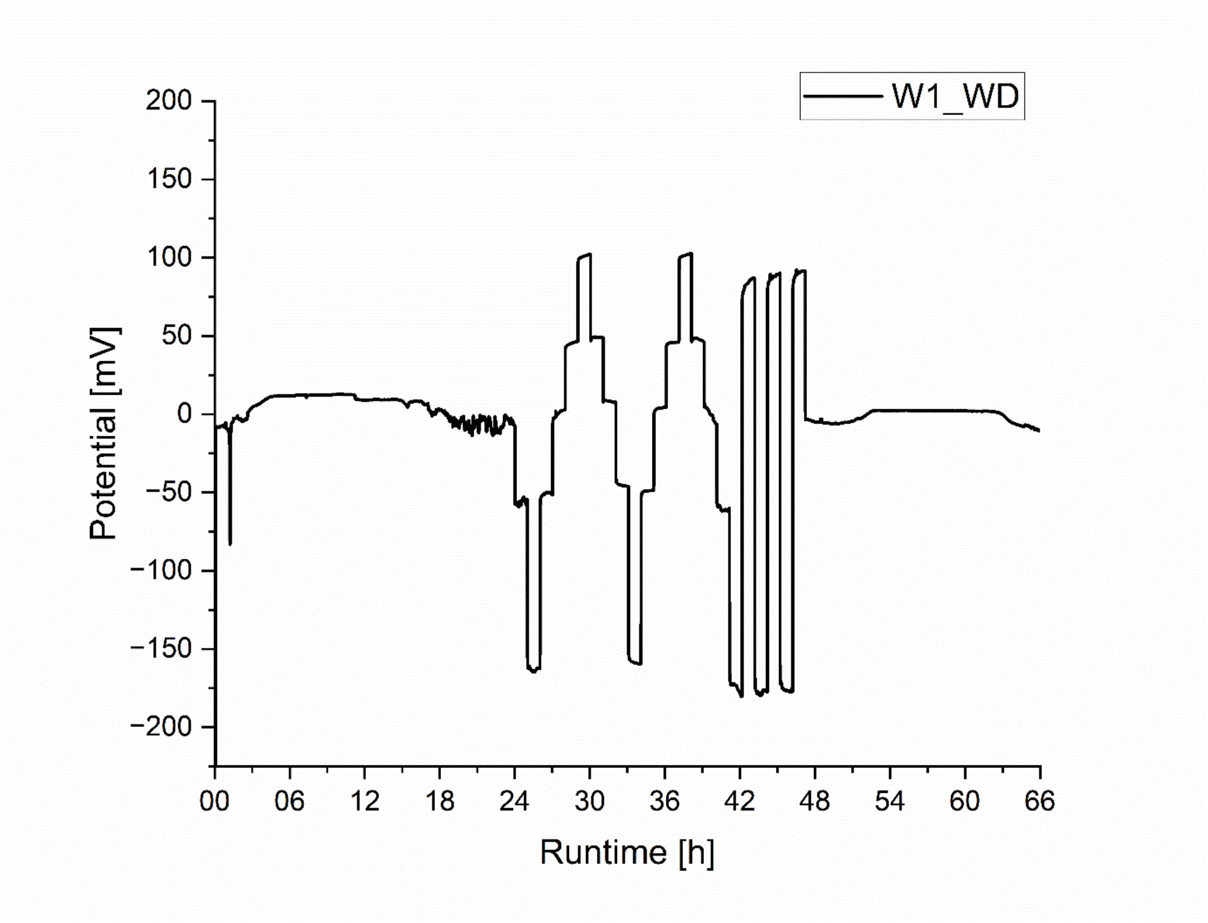

Figure 7: ISFET potential response to the 66 hours evaluation protocol with the self-made autosampler of W1 with WD in continuous measurement.

Sentron FETs with Sentron Device *OnOff*

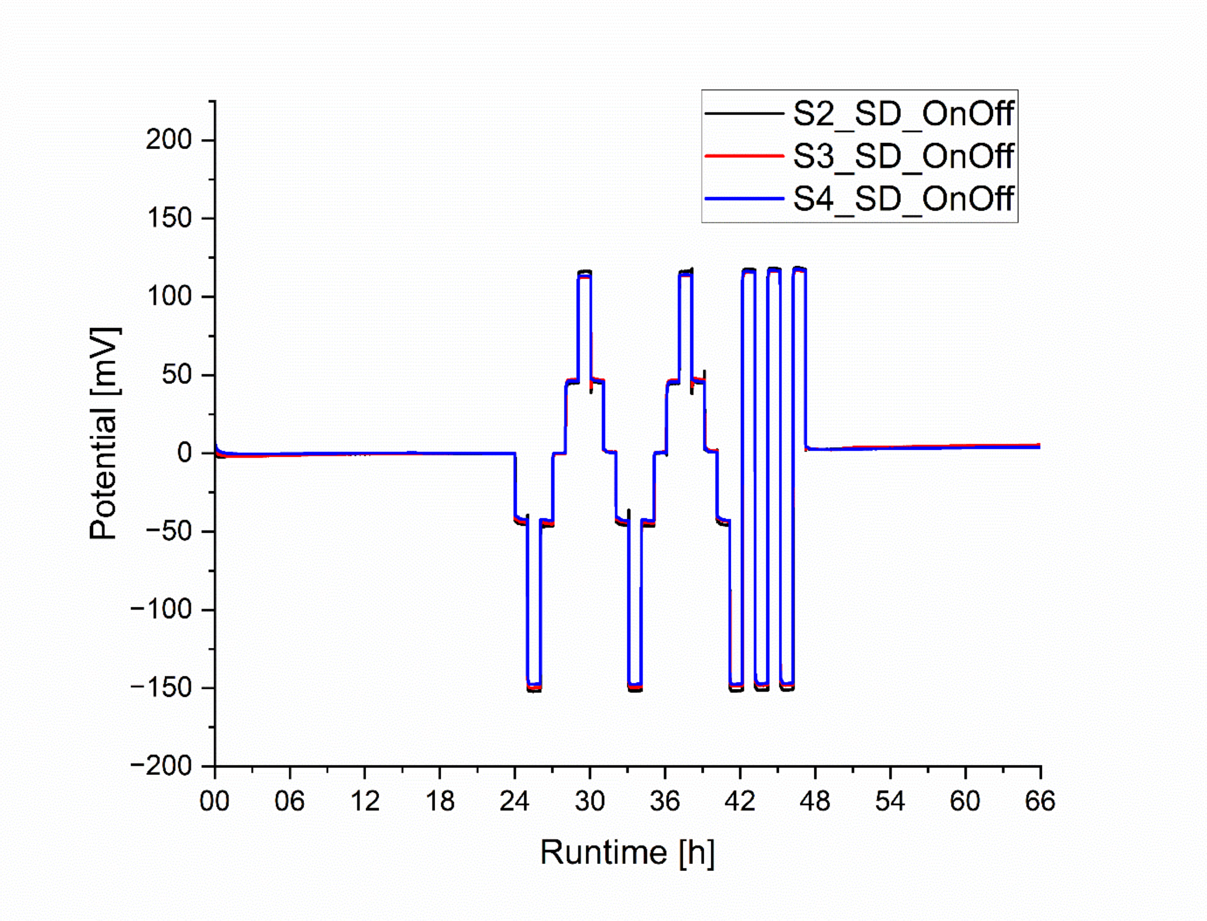

Figure 8: The stacked ISFET potential response to the 66 hours evaluation protocol with the self-made autosampler of S2, S3 and S4 with SD in OnOff-measurement.

Microsens FETs with Sentron Device *OnOff*

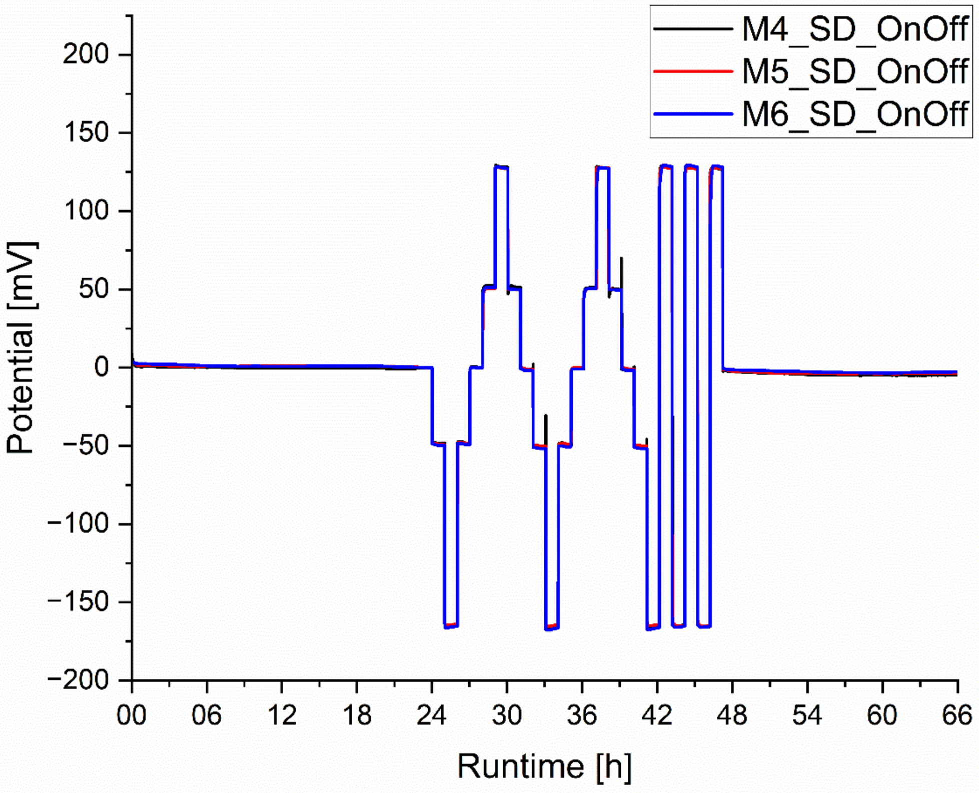

Figure 9: The stacked ISFET potential response to the 66 hours evaluation protocol with the self-made autosampler of M4, M5 and M6 with SD in OnOff-measurement.

Sentron FETs with Winsense Device *OnOff*

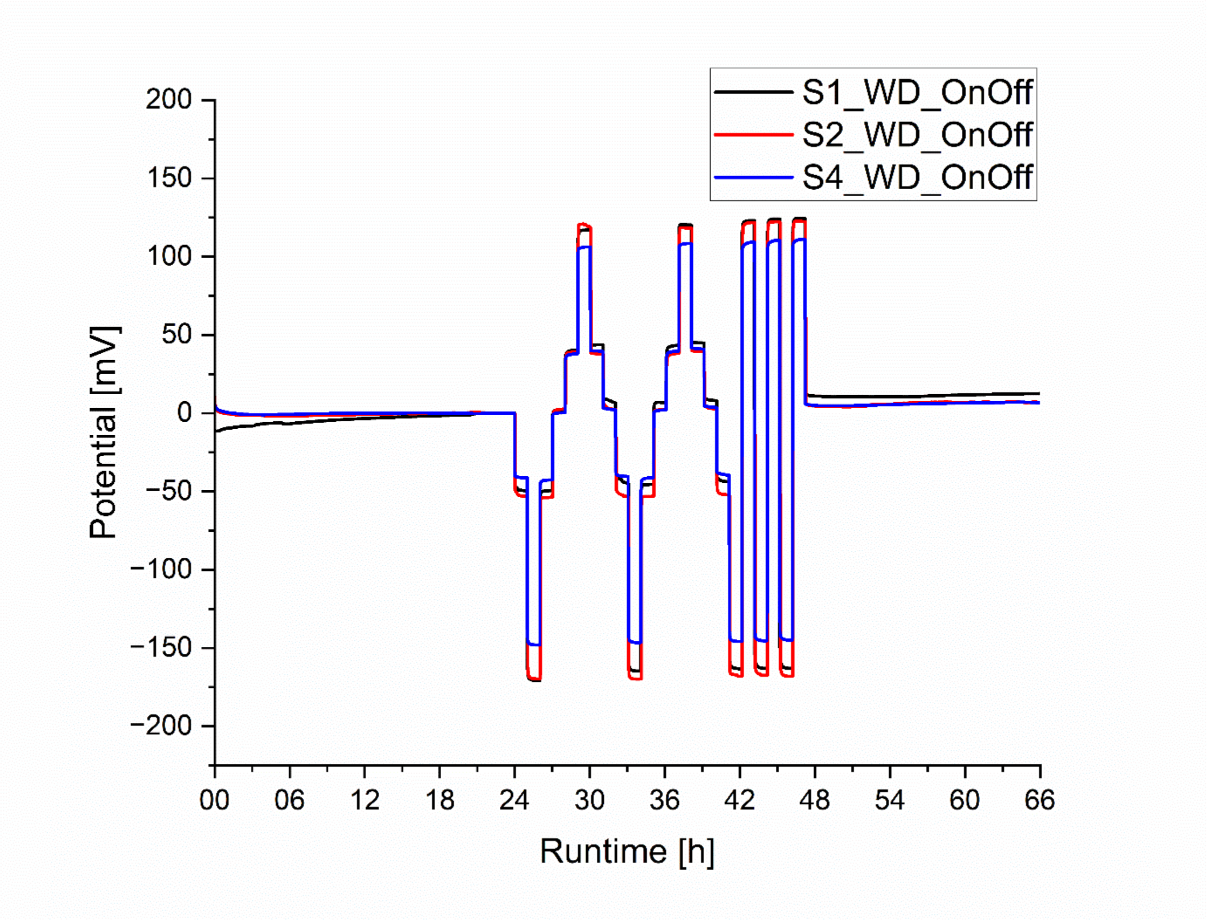

Figure 10: The stacked ISFET potential response to the 66 hours evaluation protocol with the self-made autosampler of S1, S2 and S4 with WD in OnOff-measurement.

Microsens FETs with Winsense Device *OnOff 66 h*

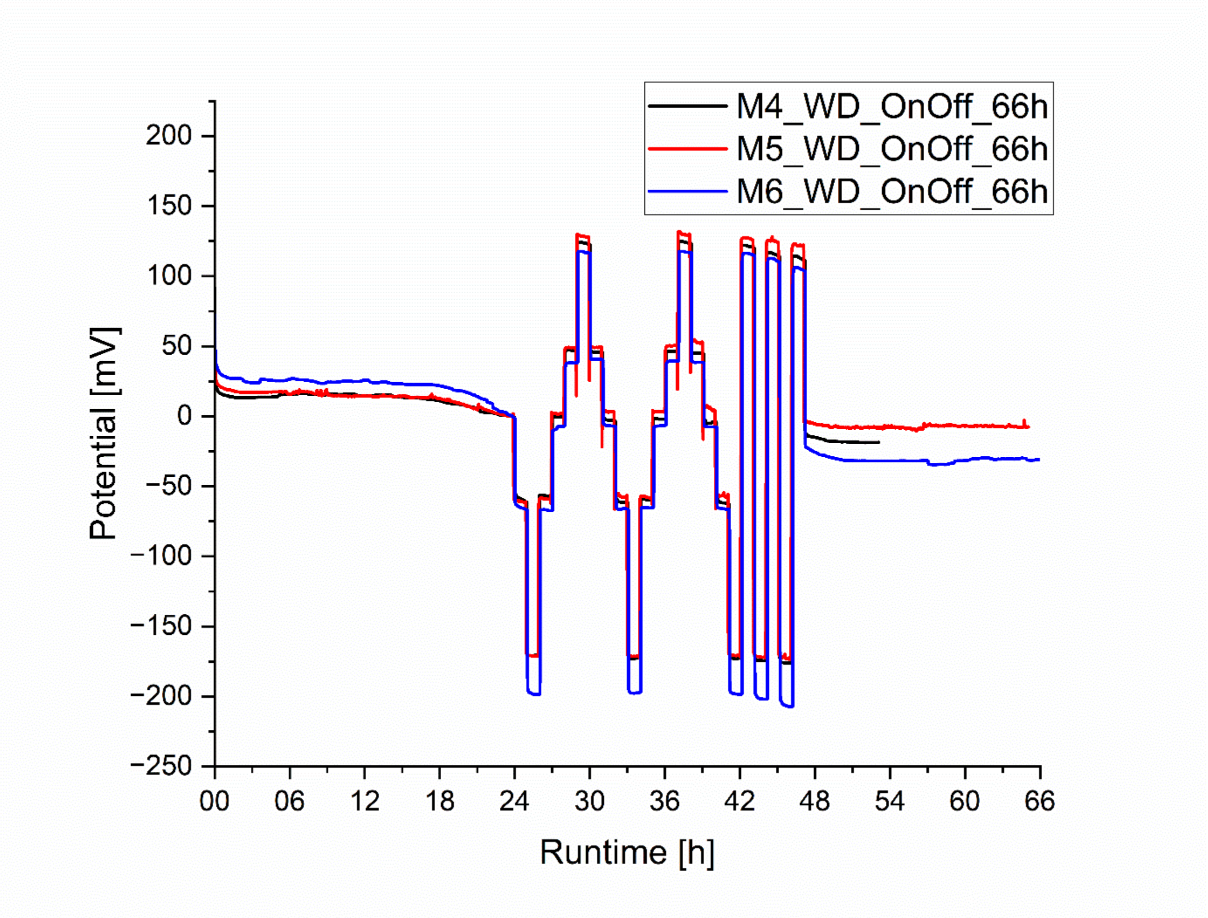

Figure 11: The stacked ISFET potential response to the 66 hours evaluation protocol with the self-made autosampler of M4, M5 and M6 with WD in OnOff-measurement.

Microsens FETs with Winsense Device *OnOff 24 h*

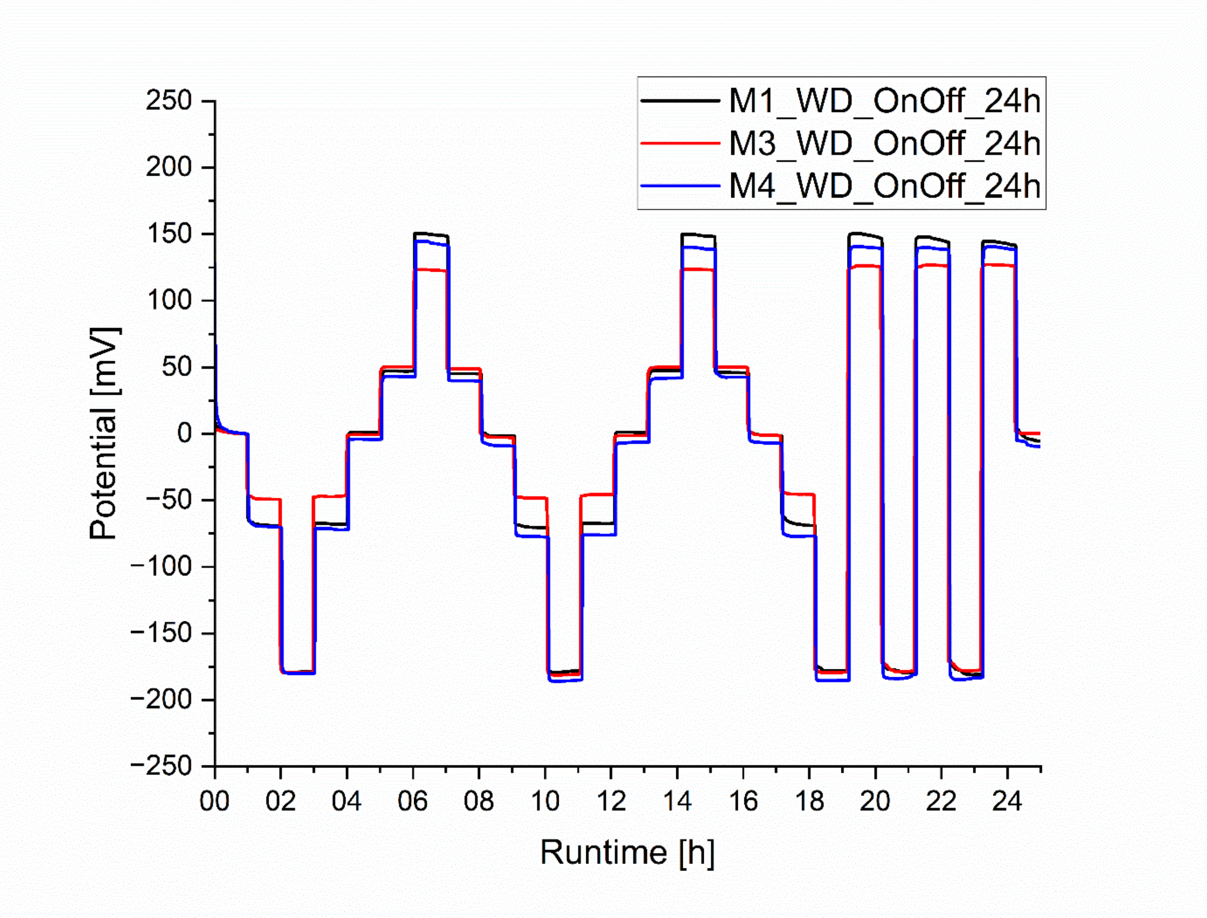

Figure 12: The stacked ISFET potential response to a 24-hour evaluation protocol with the self-made autosampler of M1, M3 and M4 with WD in OnOff-measurement.

Winsense FETs with Winsense Device OnOff

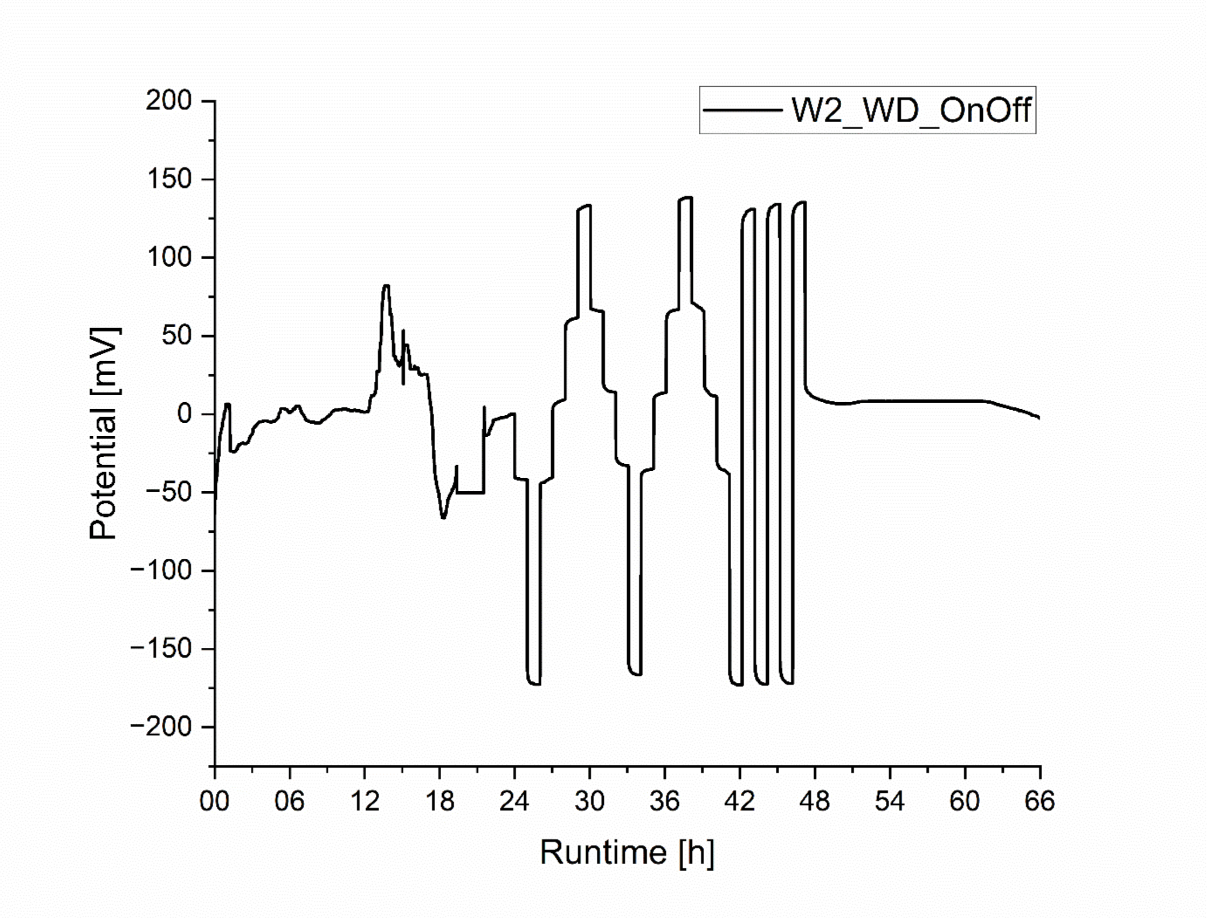

Figure 13: ISFET potential response to the 66 hours evaluation protocol with the self-made autosampler of W2 with WD in OnOff-measurement.
